# Progression of albumin/creatinine ratio (ACR) and estimated glomerular filtration rate (eGFR) over 24 years in people with type 2 diabetes. Drivers, potential protectors and associated mortality

**DOI:** 10.1111/dme.70193

**Published:** 2025-12-31

**Authors:** Andreas Matheou, Orestis Zavlis, Steve White, Thomas McDonnell, Alexander Warner‐Levy, John Warner‐Levy, Loren Wilkins, Hellena Habte‐Asres, Abigail Lay, Liliana Shalamanova, Martin Whyte, Martin Gibson, Philip A. Kalra, Adrian Heald

**Affiliations:** ^1^ University of Manchester Manchester UK; ^2^ University College London London UK; ^3^ University of Newcastle Newcastle UK; ^4^ Department of Renal Medicine, Salford Royal Hospital Northern Care Alliance NHS Foundation Trust Salford UK; ^5^ Kings College London London UK; ^6^ Manchester Metropolitan University Manchester UK; ^7^ Department of Diabetes and Endocrinology Salford Royal Hospital Salford UK; ^8^ University of Surrey Guildford UK

**Keywords:** cohort, complication, mortality, progression, prospective, renal, type 2 diabetes

## Abstract

**Introduction:**

The pathophysiology of chronic kidney disease (CKD) and type 2 diabetes (T2D) is multifactorial and associated with a plethora of underlying conditions and complications. Their link is reciprocal and understanding its nature, particularly over time, could improve the health of many.

**Methods:**

A prospective study was conducted to examine the development of the two main components of CKD (urine albumin/creatinine ratio (ACR) and estimated glomerular filtration rate (eGFR)) over 24 years (2001–2024) in a sample of 718 individuals with a diagnosis of T2D. Longitudinal modelling was conducted to examine the rate of change of ACR and eGFR over the 24 years, as well as whether sex, smoking status, glycated haemoglobin (HbA1c), systolic blood pressure (SBP), diastolic blood pressure (DBP), body mass index (BMI) influenced that rate of change in both our total sample and three sub‐groups (no CKD, CKD with increased ACR and preserved eGFR, and CKD with increased ACR and reduced eGFR).

**Results:**

At baseline, 428 (59.6%) patients were male while 290 (40.4%) were female. Mean age at baseline was 56.6 ± 12.4 years. Mean follow‐up period was 16.4 ± 2.1 years. 451 (62.8%) patients had a normal ACR and eGFR ≥60 mL/min/1.73 m^2^, no CKD. At 24‐year follow‐up, 196 (43%) of these patients had progressed to an ACR >3 mg/mmol and/or eGFR<60 mL/min/1.73 m^2^, developing CKD. At final follow‐up, 282 patients were still alive. In the whole cohort, 10 (1.4%) patients progressed to end‐stage kidney disease eGFR<15 mL/min/1.73 m^2^.

For the whole cohort ACR increased exponentially, while eGFR decreased linearly by 1.02 mL/min/1.73 m^2^ per year.

For ACR: SBP (*β* = 0.36, 95% CI [0.24, 0.48]) and DBP (*β* = 0.40, 95% CI [0.16, 0.64]) were the only significant independent predictors of ACR progression particularly in the sub‐group with increased ACR and preserved eGFR.

For eGFR: Female sex (*β* = −3.79, 95% CI [1.96, 5.63]), SBP (*β* = −0.12, 95% CI [−0.17, −0.06]), DBP (*β* = −0.19, 95% CI [−0.08, −0.31]), HbA1c (*β* = −1.17, 95% CI [−0.63, −1.71]), baseline cholesterol (*β* = 0.86, 95% CI [0.29, 1.43]) and smoking (*β* = −2.05, 95% CI [−3.80, −1.30]) were significant independent predictors of eGFR progression, but only in the non‐CKD at baseline sub‐group.

At the end of follow‐up 436 (60%) of people had died including 219 (48.6%) of the patients with no CKD at baseline, compared to 158 (76.7%) of people with increased urine ACR/preserved eGFR and 59 (96.7%) of those with increased urine ACR and reduced eGFR, with 10 year mortality rates of 6.6%, 14.5% and 26.6%, respectively. In the whole cohort only 10 (1.4%) patients progressed to end‐stage kidney disease (eGFR<15 mL/min/1.73 m^2^).

**Conclusion:**

This study revealed several factors that are associated with accelerated progression of CKD over 20 + years, including female sex and current/previous smoking. At baseline, the group with ACR >3 mg/mmol exhibited the highest rate of ACR increase. Multiple factors influenced eGFR decrease in those with baseline eGFR ≥60 mL/min/1.73 m^2^. Mortality rate was profoundly influenced by historical CKD status.

**What's new?**

**What is already known?**

- Chronic kidney disease (CKD) is a common, often silent complication of type 2 diabetes, and the presence of albuminuria and/or reduced eGFR is strongly linked to higher long‐term mortality.

**What this study has found?**

- Over 24 years in a T2D cohort, albumin: creatinine ratio (ACR) rose exponentially while eGFR declined linearly, with baseline CKD severity strongly stratifying progression and mortality (with blood pressure [BP] most consistently linked to worsening ACR).

**What are the implications of the study?**

- Early detection and aggressive management—especially BP control and comprehensive cardiometabolic risk reduction—should be prioritised before or at the first signs of CKD. Albuminuria can accelerate mortality and baseline combined albuminuria + low eGFR signals very high long‐term risk.

## 1 INTRODUCTION

One of the main comorbidities of type 2 diabetes (T2D) is chronic kidney disease (CKD). People with T2D have a 40% risk of developing CKD, while type 1 diabetes patients have a 30% chance of developing CKD through their lifetime.^1^ CKD is defined as a urine albumin/creatinine ratio (ACR) >3 mg/mmol and/or estimated glomerular filtration rate (eGFR) <60 mL/min/1.73 m^2^. CKD is generally clinically silent until the advanced stages of disease. The presence of CKD in association with T2D is associated with markedly increased mortality risk^2^, ^3^, ^4^ and, if other causes are excluded, is termed diabetic kidney disease (DKD).

CKD and T2D are closely related and being diagnosed with either one increases the probability of being diagnosed with the other in the years thereafter.^5^, ^6^ As described in the cohort study of Tao et al.,^7^ which included 5121 patients over a 10 year follow‐up period, divided almost evenly between male and female participants with a mean age of 55, those who developed T2D had a 1.38 times greater risk of further developing CKD, compared to those who did not have diabetes.^7^ Heald et al. also showed in a long‐term cohort that the presence of CKD was one of several conditions more prevalent in the years prior to the diagnosis of T2D than in people of the same age who did not go on to develop T2D.^6^ Likewise, in a large meta‐analysis of data from 13 countries, Fenta et al. showed that CKD is exacerbated when factors such as family history of T2D, high blood pressure and history of ‘cardiac disease’ appear in the history of a person with T2D.^8^

A better understanding of the factors that determine the development of CKD over time in people diagnosed with T2D could enable the targeting of pharmacological and other interventions before CKD becomes manifest or when eGFR has not yet declined.

In this study, we aimed to examine how CKD both developed and progressed in a large cohort of people with T2D over a period of 24 years. Overall, we had four goals. First, to assess the rate at which CKD develops over time in our entire sample, as well as the statistical trend it follows (i.e. linear versus non‐linear). Second, to examine whether (and, if so, to what extent) time‐invariant (sex, smoking, baseline cholesterol) and time‐variant (glycated haemoglobin (HbA1c), body mass index (BMI), systolic blood pressure (SBP) and diastolic blood pressure (DBP)) factors were related to the progression of CKD. Third, to examine the same effects in three sub‐groups: a non‐CKD (at baseline) sub‐group, a CKD group with preserved eGFR but increased ACR and a CKD group with reduced eGFR but increased ACR, in order to determine the relation between CKD status at a particular point in time and how this relates to the development of CKD over the coming years. Fourth, to examine the association of renal complication status at baseline to mortality at 24 year follow‐up.

## 2 METHODS

A four‐phase longitudinal study was conducted to examine the development of the two main components of CKD: ACR and eGFR. Phase 1 included years from 2001 to 2006, phase 2 from 2007 to 2012, phase 3 from 2013 to 2018 and phase 4 from 2019 to 2024. The same cohort of participants was followed across all four phases. Data included health outcome measures (ACR and eGFR), time‐invariant predictors (sex, age at diabetes diagnosis, smoking status and baseline cholesterol) and time‐variant predictors (HbA1c, systolic blood pressure, diastolic blood pressure and BMI). Time‐invariant predictors were recorded at baseline, while time‐variant predictors were available across three phases. Each outcome and covariate was represented by a single measurement per participant per 6‐year phase, giving up to four repeated data points over follow‐up. This approach avoided over‐representing participants with more frequent clinical testing and ensured comparability across individuals. Timing from recruitment within each wave was therefore aligned to the phase definition, not to ad hoc visit dates.

The cohort consisted of 718 adults with T2D, recruited sequentially and opportunistically between 2001 and 2004 from Salford Royal Foundation Trust clinics and local general practices. A favourable Ethical Opinion from the Salford Research Ethics Committee (REC Reference 2001/156) was obtained in 2001. Baseline and follow‐up data were obtained from the Salford Integrated Record^9^ and the Greater Manchester Care Record^10^. The only inclusion criterion was a diagnosis of type 2 diabetes at recruitment; no exclusion criteria were applied. Participants remained in follow‐up until death or censoring. Salford is a geographically stable population with very low emigration, and reductions in numbers over time reflect mortality only.

End‐stage kidney disease (ESKD) was defined as a sustained decrease in eGFR to <15 mL/min/1.73 m^2^, renal transplantation or maintenance renal dialysis. Analyses included all participants with at least 5 years of follow‐up.

### 2.1 Statistical analyses

All analyses were performed in R (version 4.3.1) using packages sur, dplyr, tidyr, naniar and mice for data preparation and imputation and panelr, lme4 and ggplot2 for modelling and visualisation. Descriptive statistics were calculated for baseline demographic and clinical characteristics (Table 1). Missing values were assumed to be missing at random and were imputed using Gibbs sampling^11^ via the mice package.

**TABLE 1:** Descriptive statistics of the clinical cohort.

CKD status	No CKD (*N* = 451)	CKD (increased ACR, normal eGFR) (*N* = 206)	CKD (increased ACR, decreased eGFR) (*N* = 61)	Total (*N* = 718)
Sex				
Male—*n* (%)	268 (59%)	129 (63%)	31 (51%)	428 (60%)
Female—*n* (%)	183 (41%)	77 (37%)	30 (49%)	290 (40%)
Ethnicity				
White British—*n* (%)	139 (31%)	61 (30%)	19 (31%)	219 (30%)
Mixed British—*n* (%)	142 (32%)	72 (35%)	15 (25%)	229 (32%)
Other—*n* (%)	14 (3%)	3 (1%)	2 (3%)	19 (3%)
Not stated	156 (34%)	70 (34%)	25 (41%)	251 (35%)
Age (years)	55.1 ± 12.6	57.3 ± 11.4	60.8 ± 9.7	56.6 ± 12.4
Age of T2D diagnosis (years)	47.7 ± 15.7	49.2 ± 14.9	54.5 ± 15.4	48.7 ± 15.6
SBP (mmHg)	150.3 ± 20.4	156.7 ± 21.8	153.1 ± 41.9	152.3 ± 23.5
DBP (mmHg)	83.2 ± 11.3	84.6 ± 11.1	78.4 ± 20.3	83.24 ± 12.4
HbA1C (%)	6.9 ± 1.4	6.9 ± 1.7	6.4 ± 2.1	6.9 ± 1.6
HbA1c (mmol/mol)	51.9 ± 10.5	51.9 ± 12.8	46.4 ± 15.2	51.9 ± 12.0
TCB (mmol/L)	5.10 ± 1.3	5.08 ± 1.5	5.18 ± 1.1	4.99 ± 1.2
Smoker	234 (51.8%)	129 (62.6%)	31 (51.8%)	394 (54.8%)
BMI at baseline (kg/m^2^)	31.6 ± 7.04	32.7 ± 7.34	31 0.2 ± 9.79	31.9 ± 7.40
eGFR at baseline (mL/min/1.73 m^2^)	92.6 ± 18.8	87.5 ± 19.7	46.6 ± 10.5	87.2 ± 35.5
ACR at baseline (mg/mmol)	1.3 ± 0.69	8.12 ± 1.1	23.2 ± 6.2	5.4 ± 2.9
Mortality 5 years later	8 (1.6%)	6 (2.8%)	5 (8.2%)	19 (2.6%)
Mortality 10 years later	30 (6.6%)	30 (14.5%)	16 (26.6%)	76 (10.5%)
ESKD on follow‐up	2 (0.4%)	5 (2.4%)	3 (4.9%)	10 (1.4%)
Mortality 24 years later	219 (48.6%)	158 (76.7%)	59 (96.7%)	436 (60.7%)

*Note*: Mean follow‐up period was 16.4 ± 2.1 years.

### 2.2 Longitudinal modelling

Four sets of regression models were applied to examine the development and progression of ACR and eGFR. First, univariate linear models estimated the change in ACR and eGFR over time. Second, univariate exponential models were tested to allow for accelerating change. Time was modelled as measurement phase, with each phase representing a 6‐year interval; coefficients therefore represent change per 6 years. This was to ensure that each time period included at least measures of ACR and eGFR.

Model fit was compared using the Akaike Information Criterion (AIC)^12^ and the Bayesian Information Criterion (BIC).^13^, ^14^, ^15^

Third, multivariable regression models included both time‐invariant (sex, smoking and baseline cholesterol) and time‐variant (HbA1c, BMI, systolic and diastolic blood pressure) predictors to estimate their unique contributions to ACR and eGFR progression. Finally, stratified analyses were performed for three baseline CKD groups: (i) no CKD (normal ACR and eGFR ≥60), (ii) increased ACR with preserved eGFR (ACR ≥3 and eGFR ≥60) and (iii) increased ACR with reduced eGFR (ACR ≥3 and eGFR <60).

All models included random intercepts for participants to account for within‐person correlation across repeated measures.^16^

### 2.3 Survival analysis

Survival probabilities were estimated using Kaplan–Meier curves for each CKD sub‐group, with survival summarised at 5, 10 and 24 years of follow‐up.

## 3 RESULTS

Table 2 presents the demographic characteristics of the entire sample and our sub‐groups, at recruitment. Of the 718 individuals, 428 (59.6%) were male and 290 (40.4%) were female. The average age and BMI of this cohort were 56.6 ± 12.4 years and 31.9 kg/m^2^, respectively. Regarding ethnicity, most (46%) of the patients did not state their ethnic origin. Of those who did, the most common ethnicity was a mixed British background (28%), followed by white British background (25%) and ‘other backgrounds’ (1%). Mean follow‐up period was 16.4 ± 2.1 years. Baseline characteristics were compared between those with longer and shorter follow‐up, with no systematic differences being observed.

**TABLE 2:** Stratified multivariate regression results on ACR.

	Dependent variable		
	No CKD	ACR	CKD: High ACR and low eGFR
		CKD: High ACR and normal eGFR	
	(1)	(2)	(3)
Phase	2.079*** (0.360)	9.505*** (1.369)	56.569*** (10.479)
Female	0.315 (0.564)	0.549 (2.169)	14.748 (19.349)
BMI	0.071* (0.042)	0.017 (0.141)	2.294* (1.196)
SBP	0.014 (0.017)	0.366*** (0.063)	0.800* (0.415)
DBP	0.039 (0.035)	0.406*** (0.124)	−0.101 (0.828)
HbA1c	0.252 (0.166)	−0.506 (0.535)	−6.495 (4.594)
TCB	−0.008 (0.176)	−0.425 (0.896)	5.169 (7.539)
Smoker	0.511 (0.539)	−0.815 (2.171)	−0.094 (16.640)
Constant	−10.711*** (3.559)	−19.833 (12.508)	−233.959** (99.893)
Observations	1353	618	183
*R* ^2^	0.039	0.117	0.170
Adjusted *R* ^2^	0.033	0.106	0.131
Residual SE	9.722 (df = 1344)	25.156 (df = 609)	105.633 (df = 174)
*F* statistic	6.809*** (df = 8; 1344)	10.117*** (df = 8; 609)	4.444*** (df = 8; 174)

Abbreviations: BMI, body mass index mean over time; DBP, Diastolic blood pressure mean over time; HbA1c, Glycated haemoglobin mean over time; SBP, Systolic blood pressure mean over time; TCB, Total cholesterol at baseline.

* *p* < 0.1.

** *p* < 0.05.

*** *p* < 0.01.

**FIGURE 1:**
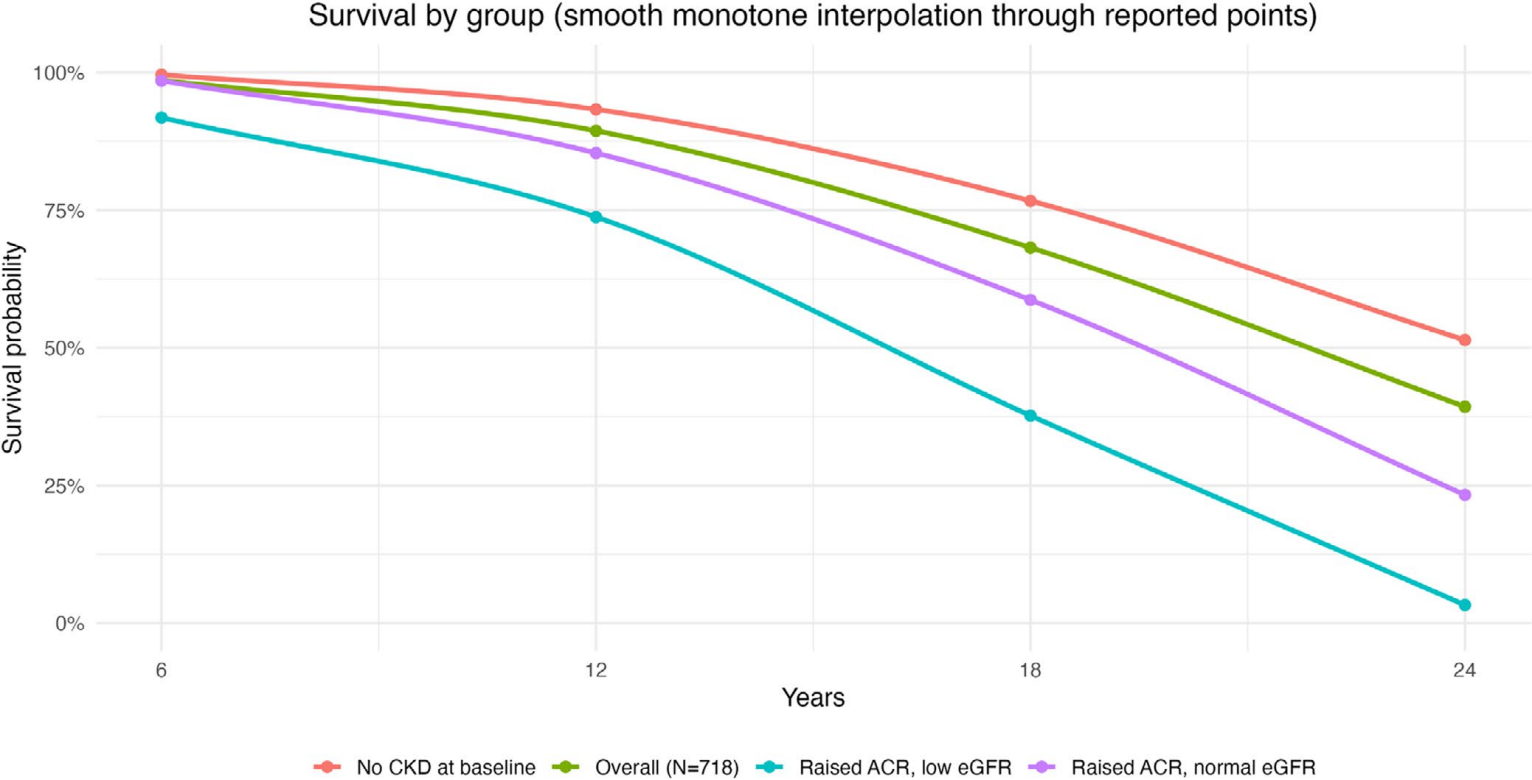
Kaplan–Meier survival curves for each of the 3 Groups as defined at baseline. Group 1 (no CKD); Group 2 (CKD with preserved eGFR but elevated ACR); Group 3 (CKD with reduced eGFR and elevated ACR).

Overall, 10 patients developed end‐stage kidney disease (ESKD) at follow‐up: 2 in the non‐CKD group, 5 in those with increased ACR and preserved eGFR and 3 in those with increased ACR and reduced eGFR. The 5‐year survival risk was relatively high for all groups: specifically, 98.4% in the non‐CKD group, 97.2% in the CKD group with increased ACR and preserved eGFR and 91.8% in the CKD group with increased ACR and reduced eGFR. The 10‐year survival risk was similarly high for the non‐CKD group (93.3%) but was lower in the CKD group with increased ACR and preserved eGFR (85.4%) and even lower in the CKD group with increased ACR and reduced eGFR (73.8%). These estimates were obtained directly from Kaplan–Meier survival curves. Reductions in numbers over time reflected mortality only, with no withdrawal or emigration (Figure 1).

At our final follow‐up (24 years later), 282 (39.3%) individuals survived: 167 (59.2%) from an initial CKD stage 1, 113 (40%) from an initial CKD stage 2 and 2 (0.8%) from an initial CKD stage 3a. None of the patients in CKD stages 3b, 4 and 5 survived at our 24‐year follow‐up. From the 167 initial CKD stage 1 patients, 63 (37.7%) retained their stage 1 status, 83 (49.7%) switched to stage 2 and 14 (8%) switched to stage 3 status (7 exhibited missing data). From the 113 patients in CKD stage 2, 74 (65.4%) retained their status and 36 (31.8%) switched to stage 3 (with 3 having missing data). Finally, from the 2 patients in CKD stage 3a, 1 remained in stage 3 while another transitioned to stage 4.

### 3.1 The progression of ACR from 2001 to 2024

In order to understand the progression of ACR over time, three regression analyses were conducted. First, a univariate regression on the entire sample (*N* = 718) indicated that ACR increased by 5.37 mg/mmol per 6 years (*β* = 5.37, CI = [4.14, 6.61], *p* < 0.001). Coefficients therefore represent change per phase, not per year. Although this result is significant and captures the progression of ACR well, its fit indices are relatively high, indicating a suboptimal fit to data (AIC = 28645.52, BIC = 28663.41). This suboptimal fit implies that a linear trend may not capture the progression of ACR in the best way possible. To address this problem, a second regression with exponential time was fitted to the data, showing that ACR progression is best conceptualised exponentially rather than linearly: *β* = 0.26, CI = [0.22, 0.30], *p* < 0.001. Indeed, the fit indices of this exponential model were almost three times lower compared to those from the linear model (i.e. AIC = 10217.42 and BIC = 10235.3), indicating a notably better fit to the data. Exponential effects are reported as percentage change per 6‐year phase to aid clinical interpretation. Altogether, these results suggest that ACR progresses exponentially over time.

**FIGURE 2:**
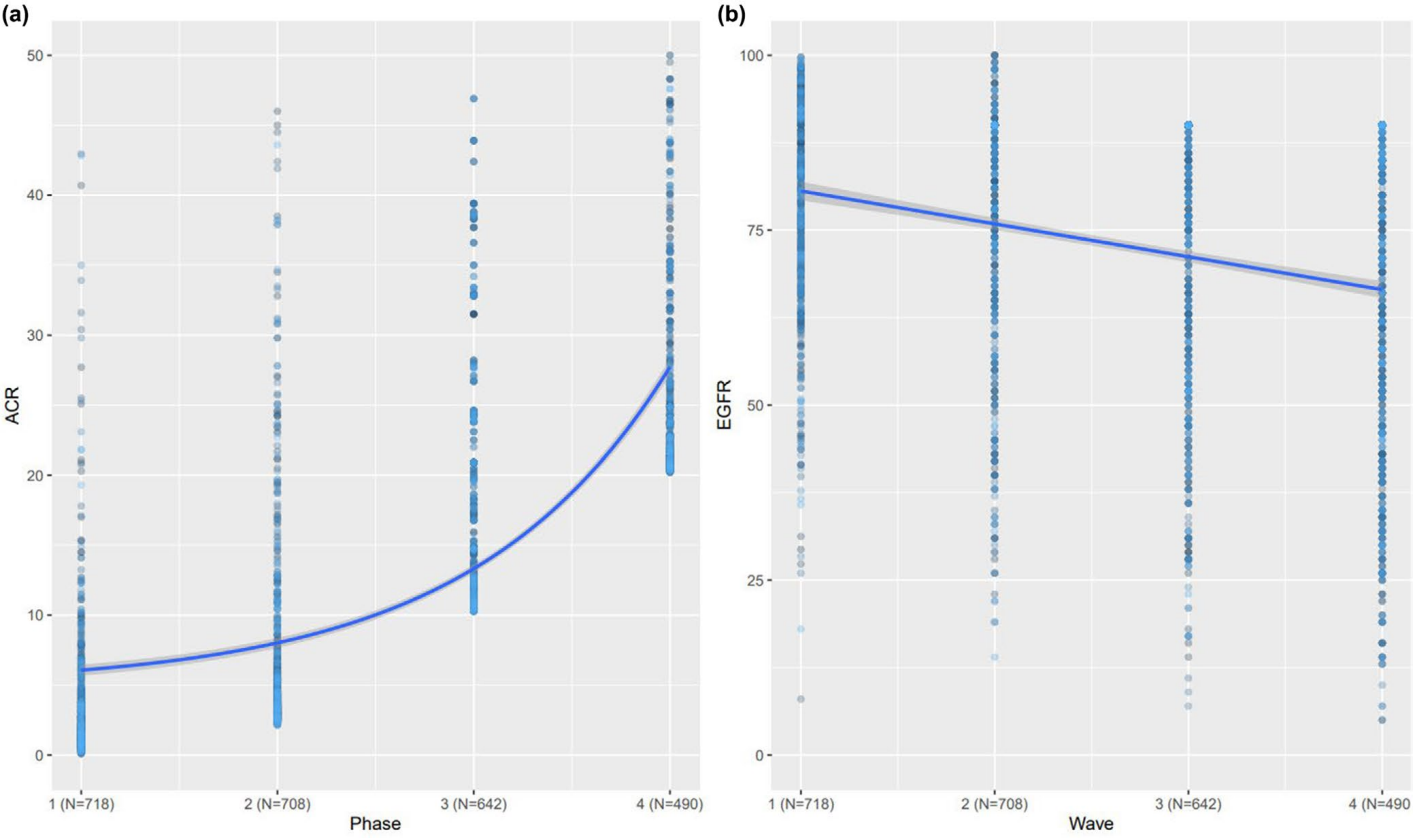
Progression of ACR and eGFR over time. Left panel (a) depicts the progression of ACR in exponential terms, which was the model that fitted the data best. Right panel (b) represents the decay of EGFR in linear terms, which was the best‐fitting decay term suggesting that eGFR's rate of change was constant over time. Right panel (b) depicts the progression of eGFR in linear terms (decrease over time).

The exponential increase of ACR was also reflected in our stratified univariate analyses, which revealed that ACR increased at an increasing rate across our three sub‐groups. Specifically, ACR increased at a low rate in the patients with no baseline CKD (*β* = 2.95, 95% CI [2.33, 3.57], *p* < 0.001), a moderate rate in the CKD group with increased baseline ACR and preserved eGFR (*β* = 7.34, 95% CI [5.49, 9.19], *p* < 0.001) and at a high rate in the CKD group with increased ACR and reduced eGFR at baseline (*β* = 11.99, 95% CI [1.26, 22.72], *p* = 0.02). Interpreted clinically, this means that patients with more severe CKD at baseline experience much faster accumulation of albuminuria over each 6‐year period (Figure 2a).

### 3.2 The progression of eGFR from 2001 to 2024

A similar analytical procedure was adopted to understand the progression of eGFR. In particular, the first regression analysis was linear and revealed that in the whole cohort eGFR decreased by 7.01 mL/min/1.73 m^2^ per 6 years (*β* = −7.01, CI = [−9.36, −4.66], *p* < 0.001). The fit indices here were relatively high (AIC = 32700.57 and BIC = 32718.46), but still indicated good fit. To examine whether this fit improved with a more complex model, an exponential regression was conducted, indicating that, unlike ACR, eGFR progression was not well accounted for by a putative non‐linear decay: *β* = −0.11, SE = 0.36, CI = [−0.12, −0.09], *p* = 0.04, AIC = 43264.88 and BIC = 43283.48. These patterns imply that, unlike ACR, eGFR's temporal decay occurs linearly, not exponentially.

A set of stratified univariate analyses revealed a similar pattern by showing that eGFR decreased at a similar rate across our three sub‐groups. Specifically, eGFR decreased at a moderate rate in the sub‐group with no baseline CKD (*β* = −7.90, 95% CI [−8.61, −7.19], *p* < 0.001), with a similar but not statistically significant rate of change in the CKD group with increased ACR and preserved eGFR at baseline (*β* = −7.93, 95% CI [−15.87, 8.16], *p* > 0.05) and a slightly decreased rate in the CKD group with increased baseline ACR and reduced eGFR (*β* = −5.62, 95% CI [−7.03, −4.20], *p* < 0.001). This confirms that eGFR declines linearly over time, at similar rates across clinical groups (Figure 2b).

### 3.3 Factors influencing the progression of ACR

**FIGURE 3:**
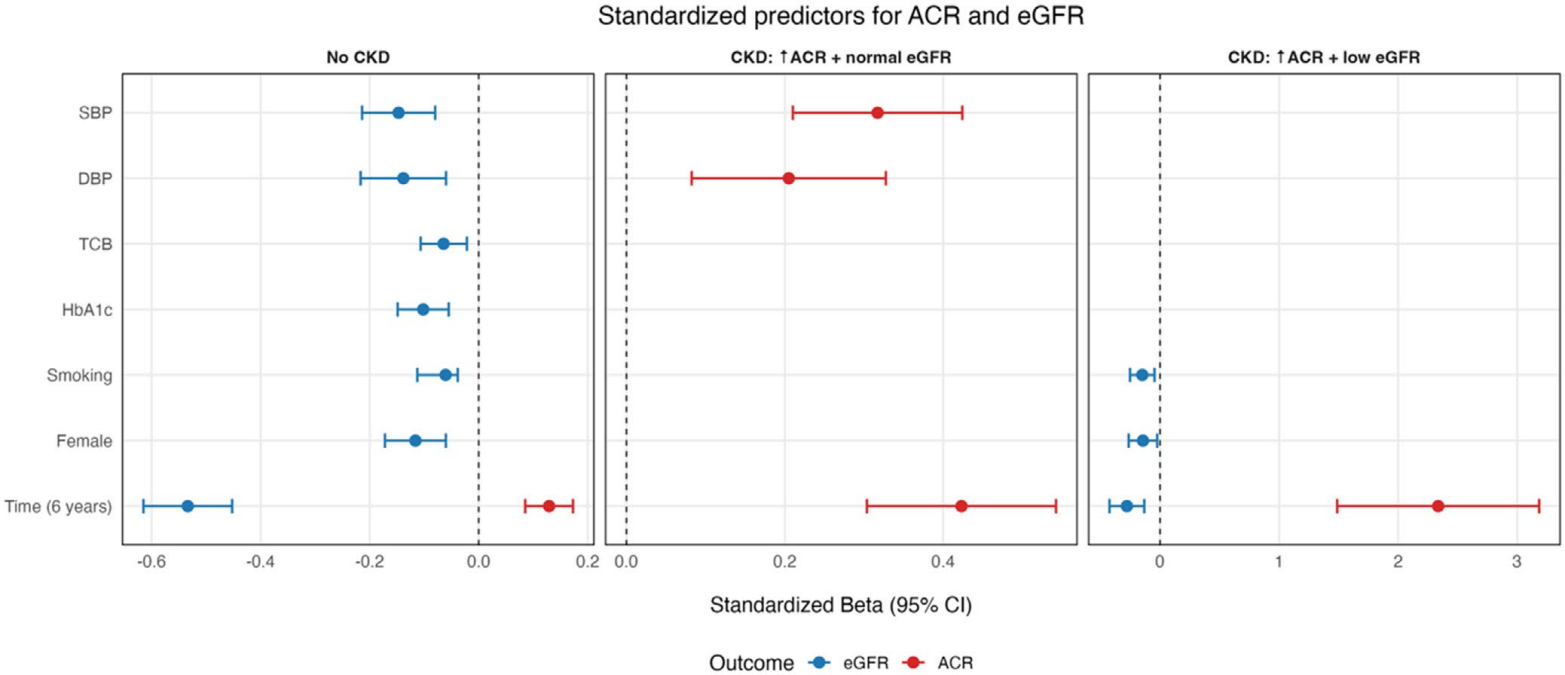
Forest Plot for the regression effects (beta coefficients represented by circles and confidence intervals by the width of lines) on the whole dataset phases and time‐variant and time‐invarianttime invariant variables.

Beyond time effects, we conducted multivariable analyses (on both the total sample and our three sub‐groups) to examine the unique effects of time, sex, BMI, systolic and diastolic blood pressure, HbA1c and baseline cholesterol levels on ACR progression. At the total sample level, these analyses revealed two significant effects: the unique effects of time (*β* = 7.08, CI = [5.25, 8.91], *p* < 0.001) and systolic blood pressure on ACR progression (*β* = 0.24, CI = [0.16, 0.32], *p* < 0.001). At the sub‐group level, the unique effect of time survived, showing that ACR increases at an increasing rate across the three sub‐groups: specifically, the non‐CKD sub‐group exhibited the slowest time trend (*β* = 2.07, 95% CI [1.37, 2.78], *p* < 0.001), the CKD sub‐group with baseline increased ACR and preserved eGFR exhibited a moderate time trend (*β* = 9.50, 95% CI [6.81, 12.19], *p* < 0.001) and the CKD group with increased ACR and decreased eGFR at baseline exhibited the fastest time trend (*β* = 56.56, 95% CI [35.88, 77.25], *p* < 0.001). We now report these as change per 6‐year phase to aid clarity. Beyond time effects, the only other (Bonferroni‐corrected) significant effects on ACR progression were systolic blood pressure (*β* = 0.36, 95% CI [0.24, 0.48], *p* < 0.001) and diastolic blood pressure (*β* = −0.40, 95% CI [−0.64, −0.16], *p* = 0.001), which were only evident in the CKD sample with increased ACR and preserved eGFR (see Figure 3).

### 3.4 Factors influencing the progression of eGFR

Repeating the same multivariable analyses for eGFR, we found different patterns (Figure 2). First, we found no significant multivariate effects on our total sample. We did find seven independent significant effects in the non‐CKD sub‐group: time (*β* = −7.67, 95% CI [−8.84, −6.50], *p* < 0.001), female sex (*β* = −3.79, 95% CI [−5.63, −1.96], *p* < 0.001), systolic blood pressure (*β* = −0.12, 95% CI [−0.17, −0.06], *p* < 0.001), diastolic blood pressure (*β* = −0.19, 95% CI [−0.31, −0.08], *p* < 0.001), HbA1c (*β* = −1.17, 95% CI [−1.71, −0.63], *p* < 0.001), baseline cholesterol level (*β* = −0.86, 95% CI [−1.43, −0.29], *p* < 0.001) and smoking (*β* = −2.05, 95% CI [−3.80, −1.30], *p* = 0.001). Finally, we found two significant effects in the CKD sub‐group with baseline increased ACR and decreased eGFR: namely, the effect of time (*β* = −4.78, 95% CI [−7.31, −2.26], *p* < 0.001) and smoking (*β* = −5.82, 95% CI [−9.83, −1.81], *p* < 0.001). For the group with raised ACR and normal GFR, no significant associations were found with a change in eGFR. In multivariate models including both ACR and eGFR, no cross‐predictive effects were observed, indicating that their trajectories were statistically independent. These patterns imply that time and smoking status were the most robust predictors of eGFR decrease over time in the non‐CKD sub‐group and the CKD sub‐group with increased ACR and reduced eGFR, while multiple factors were associated with eGFR decline in those with no CKD at baseline (see Figure 3 for a visual outline).

## 4 DISCUSSION

The mitigation of cardiovascular disease, mortality risk and the progression of CKD are pivotal to CKD management in individuals with T2D, regardless of whether the underlying renal dysfunction is the direct result of the diabetes environment or is superimposed on T2D. In this study, we have described the progression of ACR and eGFR in individuals with T2D, over 24 years. We report one of the longest follow‐up periods of people with T2D in relation to determination of renal function change over time. Our results indicate that while ACR increases exponentially over time, eGFR does so linearly. In multivariate models including both ACR and eGFR, no cross‐predictive effects were observed, indicating that their trajectories were statistically independent.

We accept that as time progresses, patients with T2D are more likely to have a higher ACR.^1^ However, what we are examining here is how the characteristics of specific sub‐groups based on CKD status at baseline relate to the rate of increase in ACR or of decrease in eGFR within the overall group of people with T2D for whom, as for the general population, eGFR declines over time.

It is notable that only a small number of variables were associated with ACR progression, while a broader range were linked to eGFR decline. This may reflect the fact that ACR is predominantly driven by haemodynamic factors such as blood pressure, whereas eGFR decline is affected by multiple pathophysiological processes including glycaemic control, lipid status, sex and smoking.

The analysis also showed how CKD status more than 24 years in the past is associated with prospective mortality rates in people with T2D. The group with both raised ACR >3 mg/mmol and eGFR <60 mL/min/1.73 m^2^ showed a much high mortality rate than would be expected for the locality for people of the mean age of the participants (60.8 years)—for whom male 24 year mortality rate over the last 10 years is 49.1% and female 24 year mortality rate is 42.8%.

We accept that the mortality rates are relatively low at 5‐ and 10‐year follow‐up. However, this may relate to the age of the cohort at baseline (recruitment) being 56.6 years. The rate of development of ESKD was relatively low at the last follow‐up. This may well be a consequence of the very high mortality rate of those with CKD at baseline, with the consequence that people died before they could develop ESKD in many cases. Cardiovascular events almost certainly account for much of this excess mortality, in line with previous reports showing that as many as 70% of individuals with diabetes and CKD die from cardiovascular causes.

For progression of ACR, the greater the degree of CKD (at time of recruitment to the study) the faster the rate of increase in ACR over time, with a mitigating factor being lower systolic blood pressure and lower diastolic blood pressure for those individuals with increased ACR and normal eGFR at recruitment. The high mortality rate in those with raised ACR and eGFR under 60 mL/min/1.73 m^2^ highlights the importance of early identification and intervention with an Angiotensin‐Converting Enzyme (ACE) inhibitor or Angiotensin II Receptor Blocker (ARB), in relation to the first signs of CKD. The exponential trend for increase in ACR may relate to the way that adverse haemodynamic processes result in a rapid escalation of glomerular damage, once tissue injury occurs, with continued elevation in glomerular capillary flows and pressures predisposing to further glomerular injury.^17^

The absence of any independent relation of HbA1c with ACR progression may be linked to the relatively good glycaemic control of the cohort as a whole, at least at baseline, with a mean HbA1c for the whole cohort of 51.9 mmol/mol (6.9%) (see Table 1). This has been noted previously in a much larger cohort of people with T2D from Salford and likely relates to the historical cohesiveness of service delivery in that locality.

For eGFR, the rate of decline was similar in all three groups. The fact that higher systolic and diastolic blood pressure were associated with faster eGFR decline in people with no CKD at recruitment supports the strategy of optimisation of cardiometabolic profile and particularly lowering of blood pressure with ACE‐inhibitors or ARBs in order to have the greatest beneficial impact on future CKD rates in people with T2D. This observation has recently been reported in a review^18^ and separately in a large randomised controlled trial.^19^ The de Galan study^19^ included 11,140 people with T2D and showcased a 21% reduced risk of developing a renal disease if a fixed combination of perindopril‐Indapamide was administered before developing CKD. It should be pointed out that the ACCORD trial demonstrated that among a subset of ACCORD‐BP trial participants, intensive BP control was associated with a reduction in eGFR, but not with an increase in markers of renal injury. It was suggested that the eGFR decline observed with intensive blood pressure goals in ACCORD participants might predominantly reflect hemodynamic alterations.^20^

We identified that female sex was associated with a progressive decrease in GFR in the sub‐group of patients with no baseline CKD, indicating a greater risk of CKD in the future. Previous reports have described more rapid CKD progression in older female patients,^4^, ^21^, ^22^, ^23^ which may be due to a greater prevalence of adverse factors and/or less prescribing of cardioprotective medication. Women have also been underrepresented in clinical trials for kidney and cardiovascular protective drugs, and this is an area that needs attention.^24^

The association between smoking and decline in eGFR in the sub‐groups with no CKD at baseline and those with both raised ACR and reduced GFR is consistent with the meta‐analysis by Jiang et al.,^25^ which concluded that patients who smoke have a higher prevalence of progressive CKD in people with diabetes, along with an enhanced relative risk for all renal diseases in individuals with T2D, including microalbuminuria, macroalbuminuria and end‐stage renal disease.^26^ Smoking appears to be causative in this regard^27^ rather than just a surrogate for unhealthy lifestyle behaviours. Unfortunately, there is no immediate reversal of the risk of renal decline, immediately after quitting smoking.

In recent years, several classes of medications with renoprotective properties have been introduced for individuals with diabetes, including SGLT‐2 inhibitors, incretin receptor agonists and finerenone.^28^, ^29^, ^30^ In our cohort, prescribing of SGLT‐2 inhibitors was relatively low until the last 5 years, by which time many participants with advanced CKD had already died. Their impact on the overall trajectories is therefore likely to have been limited, with ACE‐inhibitor/ARB prescribing remaining the main reno‐protective strategy throughout most of the study period. With greater adoption of these renoprotective therapies, we may see a significant mitigation of CKD progression in patients with diabetes.

It was recently highlighted that ACR demonstrates a high degree of within‐individual variability among individuals with T2D.^31^, ^32^ However, we were able to analyse up to 15 or more serial urine samples for ACR estimation for each individual, thus minimising the impact of intraindividual variability over time.

The progressively increasing mortality rate with CKD stages, as shown in Table 3, is compatible with other studies and highlights the importance of early intervention, as does the rate of ESKD development. Numerous trials have shown that blood pressure control can reduce proteinuria and is associated with better cardiovascular outcomes in DKD.^33^ Inhibition of the Renin‐Angiotensin‐Aldosterone System with ACE inhibitors^31^ or ARB,^34^ is the preferred choice for antihypertensive medications for patients with DKD, especially for those with evidence of albuminuria. Blood pressure goals for patients with DKD may differ depending on the task force or organization from which recommendations are taken.^35^

**TABLE 3:** Stratified multivariate regression results on eGFR.

	Dependent variable		
	No CKD	EGFR	CKD: HHigh ACR and Low eGFR
		CKD: High ACR and Normal eGFR	
	(1)	(2)	(3)
Phase	−7.673*** (0.597)	22.262 (13.918)	−4.786*** (1.279)
Female	−3.799*** (0.934)	28.179 (22.041)	−5.558** (2.362)
BMI	0.086 (0.069)	1.785 (1.434)	−0.101 (0.146)
SBP	−0.116*** (0.027)	−0.165 (0.638)	−0.061 (0.051)
DBP	−0.197*** (0.057)	0.212 (1.258)	0.181* (0.101)
HbA1c	−1.171*** (0.275)	−4.419 (5.437)	0.235 (0.561)
TCB	−0.865*** (0.292)	−6.187 (9.108)	0.540 (0.920)
Smoker	−2.059** (0.893)	24.921 (22.062)	5.821*** (2.032)
Constant	84.776*** (5.897)	37.247 (127.122)	44.801*** (12.196)
Observations	1353	618	183
*R* ^2^	0.152	0.014	0.176
Adjusted *R* ^2^	0.146	0.001	0.138
Residual SE	16.107 (df = 1344)	255.666 (df = 609)	12.896 (df = 174)
F statistic	30.007*** (df = 8; 1344)	1.054 (df = 8; 609)	4.655*** (df = 8; 174)

Abbreviations: BMI, body mass index mean over time; DBP, Diastolic blood pressure mean over time; HbA1c, Glycated haemoglobin mean over time; SBP, Systolic blood pressure mean over time; TCB, Total cholesterol at baseline.

* *p* < 0.1.

** *p* < 0.05.

*** *p* < 0.01.

We found that the only independent predictors of increasing ACR were systolic and diastolic blood pressure. This does accord with the UKPDS study, where the factor most strongly associated with the development of albuminuria in type 2 diabetes was blood pressure control. Specifically, more intensive blood glucose control resulted in a 33% reduction in the relative risk of development of microalbuminuria.^36^ In the same paper, it was reported that more intensive blood glucose control resulted in a significant reduction in the proportion doubling their plasma creatinine (0.91 vs. 3.52%) at 12‐year follow‐up. Change in eGFR over time has multiple determinants, as we report and as also described in a 2021 study (38).

## 5 LIMITATIONS

Despite the strengths of this study (most notably, its longitudinal design), several limitations must also be acknowledged. First and foremost is the fact that we did not analyse the effects of ACE inhibitors or ARB. These are well‐known to alter eGFR slopes, moderate ACR and even prevent the development of nephropathy. Nevertheless, this study covers a period of 24 years during which, for many patients, despite the availability of these therapies, there was still a progression of CKD.

In order better to understand the progression of CKD (and the factors that foster it), future studies may wish to replicate the current study's design and extend it by including a non‐diabetes control group. Second, various other limitations in our dataset did not enable us to examine additional predictors. For example, ethnicity (such as African, Asian or African‐Caribbean) is a well‐known risk factor for T2D. However, the data from this cohort had either generally defined ethnic identities (i.e., mixed British without specifying the mixed racial background) or did not specify those identities (i.e., missing values).

Finally, we acknowledge that we did not have data on duration of diabetes or cause of death. The absence of diabetes duration limits our ability to assess its direct influence on trajectories. In any event, true duration is rarely available for T2D, which often has an insidious onset. However, baseline CKD severity (our stratification) partly captures cumulative disease burden and was explicitly modelled. Moreover, the lack of cause‐of‐death data precludes attributional analyses, but given the high mortality rate, it is highly likely that cardiovascular events accounted for the majority of deaths, as consistently reported in previous literature.

## 6 CONCLUSION

This cohort study revealed several factors that associate with progression of CKD over 20+ years. A much faster rate of increase in ACR was seen in individuals with elevated systolic and diastolic blood pressure at baseline. Multiple factors were associated with eGFR decrease in those with baseline eGFR ≥60 mL/min/1.73 m^2^ including female sex, elevated systolic and diastolic blood pressure at baseline and HbA1c.

The 97% 24 year mortality rate in those with raised ACR >3 mg/mmol and eGFR under 60 mL/min/1.73 m^2^ at baseline (much higher than the background mortality rate for people of a similar age) and lower mortality rate in those with normal ACR or eGFR highlights the importance of early identification and intervention in relation to the first signs of CKD.

## AUTHOR CONTRIBUTIONS

AH and MG conceived the study and designed the protocol in consultation with PK. AM, JWL, AWL and LW extracted and anonymized the data; AM and OZ performed data processing and data analysis in consultation with TM. PK and AH. SW, LS, AL and HHA provided scientific context together with MG, MW and PK, who also provided senior review. All authors reviewed the manuscript during its development, approved the final version and agree to be accountable for all aspects of the work.

## FUNDING INFORMATION

This research did not receive any specific grant from funding agencies in the public, commercial or not‐for‐profit sectors.

## CONFLICT OF INTEREST STATEMENT

None of the authors have any conflicts of interest to declare.

## ETHICS STATEMENT

A favourable ethical opinion was given by Salford Research Ethics Committee (REC) Ref 2001/156 in 2001.

## GUARANTOR STATEMENT

AH is the guarantor of this work and, as such, had full access to all the data in the study and takes responsibility for the integrity of the data and the accuracy of the data analysis.

## Data Availability

The data that support the findings of this study are available on request from the corresponding author. The data are not publicly available due to ethical restrictions.
